# Analysis of common genetic variation across targets of microRNAs dysregulated both in ASD and epilepsy reveals negative correlation

**DOI:** 10.3389/fgene.2023.1072563

**Published:** 2023-03-09

**Authors:** Carol Stella, Covadonga M. Díaz-Caneja, Maria Jose Penzol, Alicia García-Alcón, Andrea Solís, Álvaro Andreu-Bernabeu, Xaquín Gurriarán, Celso Arango, Mara Parellada, Javier González-Peñas

**Affiliations:** ^1^ Department of Child and Adolescent Psychiatry, Institute of Psychiatry and Mental Health, Hospital General Universitario Gregorio Marañón, Madrid, Spain; ^2^ Instituto de Investigación Sanitaria Gregorio Marañón (IiSGM), Madrid, Spain; ^3^ CIBERSAM, Centro Investigación Biomédica en Red Salud Mental, Instituto de Salud Carlos III, Madrid, Spain; ^4^ School of Medicine, Universidad Complutense, Madrid, Spain

**Keywords:** autism, epilepsy, microRNA, comorbidity, genetic correlation, genetic heterogeneity

## Abstract

Genetic overlap involving rare disrupting mutations may contribute to high comorbidity rates between autism spectrum disorders and epilepsy. Despite their polygenic nature, genome-wide association studies have not reported a significant contribution of common genetic variation to comorbidity between both conditions. Analysis of common genetic variation affecting specific shared pathways such as miRNA dysregulation could help to elucidate the polygenic mechanisms underlying comorbidity between autism spectrum disorders and epilepsy. We evaluated here the role of common predisposing variation to autism spectrum disorders and epilepsy across target genes of 14 miRNAs selected through bibliographic research as being dysregulated in both disorders. We considered 4,581 target genes from various *in silico* sources. We described negative genetic correlation between autism spectrum disorders and epilepsy across variants located within target genes of the 14 miRNAs selected (*p* = 0.0228). Moreover, polygenic transmission disequilibrium test on an independent cohort of autism spectrum disorders trios (N = 233) revealed an under-transmission of autism spectrum disorders predisposing alleles within miRNAs’ target genes across autism spectrum disorders trios without comorbid epilepsy, thus reinforcing the negative relationship at the common genetic variation between both traits. Our study provides evidence of a negative relationship between autism spectrum disorders and epilepsy at the common genetic variation level that becomes more evident when focusing on the miRNA regulatory networks, which contrasts with observed clinical comorbidity and results from rare variation studies. Our findings may help to conceptualize the genetic heterogeneity and the comorbidity with epilepsy in autism spectrum disorders.

## Introduction

Autism spectrum disorder (ASD) comprises a group of neurodevelopmental disorders characterized by impairments in social interaction and communication, restricted interests and repetitive behaviors (Bourgeron, 2015) that affect around 1% (Zeidan et al., 2022) and 2.3% (Maenner, 2021) of the population worldwide. ASD displays great clinical heterogeneity and shows high comorbidity rates with neuropsychiatric and other medical conditions (Tye et al., 2019). Epilepsy is a severe neurological condition characterized by recurrent seizures with 0.76% of lifetime prevalence across the worldwide population (Fiest et al., 2017).

The co-occurrence of ASD and epilepsy has been well documented at the epidemiological level: between 11% and 39% of ASD patients have epilepsy (Canitano, 2007; Lukmanji et al., 2019), while around 15%–47% of people with epilepsy also have ASD (Clarke et al., 2005; Lukmanji et al., 2019). This comorbidity is also associated with intellectual disability and greater severity of the ASD symptomatology (Ko et al., 2016; Lee et al., 2020). Beyond clinical comorbidity, a clear pattern of biological overlap between ASD and epilepsy has been demonstrated (Lee et al., 2015). At the cellular level, several studies have suggested that an alteration of the excitatory—inhibitory (E/I) balance could underlie this co-occurrence (Frye and Rossignol, 2016; Bozzi et al., 2018).

From the genetic perspective, both ASD and epilepsy have been described as complex disorders with considerable heritability estimates (around 80% for ASD (Bai et al., 2019) and 32% for epilepsy (Chen et al., 2017)), which denotes a solid genetic base. A consistent polygenic contribution from both common (Abou-Khalil et al., 2018; Grove et al., 2019) and rare protein-disrupting variation (Feng et al., 2019; Satterstrom et al., 2020; Motelow et al., 2021) has been reported for both ASD and epilepsy. Previous studies have evidenced the genetic overlap between both disorders at the rare variant level; ASD patients with rare protein-disrupting variants, especially affecting neurodevelopmental genes, show increased likelihood of comorbid epilepsy (Bishop et al., 2021). This has been observed for some genetic syndromes such as Phelan-McDermid (Bozzi et al., 2018) or the Fragile X syndrome (Chen et al., 2017) but it has also been described at a genome-wide level for *de novo* disrupting mutations (Heyne et al., 2018; Satterstrom et al., 2020; Mahjani et al., 2021). In fact, the diagnostic yield of reportable pathogenic variants in ASD is increased from 5%–19% to 15%–60% when comorbid epilepsy is present (Bishop et al., 2021).

By contrast, at the common genetic variation level, recent studies have suggested a limited shared genetic risk from common polygenic contributions across both disorders (The Brainstorm Consortium et al., 2018). However, existent genetic correlation between complex traits may reside in specific pathways and remain undetected when analyzing the whole common genetic variation across the genome (Colbert et al., 2021; Perry et al., 2022). In the past few years, new genomic tools have been developed in order to study the shared common variation in specific genome regions or pathways. Methods such as p-HESS (Shi et al., 2017) or GNOVA (Lu et al., 2017) allow to estimate partial genetic correlation and annotation-stratified covariance to unravel potential specific mechanisms involved in the comorbidity between disorders*.*

Dysregulation of microRNAs (miRNAs), non-coding RNAs that regulate gene expression by repressing translation *via* destabilization of mRNA species, has been consistently described in both ASD and epilepsy (Issler and Chen, 2015; Toma et al., 2015; Wu et al., 2016; Wang and Zhao, 2021). MiRNAs regulate the expression of key genes during neurodevelopment and adult brain structure and function, by acting on processes such as synaptogenesis, neurogenesis, neuronal differentiation, and neuroplasticity. Some specific miRNAs have been described to be down- or up-regulated both in ASD and epilepsy. For instance, miR-146a has been found to be upregulated in ASD children and patients with epilepsy (Wang et al., 2015; An et al., 2016; Nguyen et al., 2018). Another example is miR-199, which has been found to be upregulated in patients with temporal lobe epilepsy with hippocampal sclerosis (Antônio et al., 2019), but downregulated in ASD (Sarachana et al., 2010). Since many of these miRNAs are dysregulated both in ASD and other neurodevelopmental disorders (Hicks and Middleton, 2016; Hu et al., 2017), a combination of miRNAs may be potentially used as novel biomarkers for ASD diagnosis or even to describe subgroups of ASD (Hicks et al., 2020; Cui et al., 2021; Salloum-Asfar et al., 2021). Similarly, the potential use of miRNAs as biomarkers and therapeutic targets for epilepsy has been suggested (Henshall et al., 2016; Wang and Zhao, 2021).

Collectively, miRNAs are predicted to regulate around the 60% of the protein-coding genes, by establishing complex regulatory pathways for each miRNA (Friedman et al., 2009). The implication of common genetic variation within genes targeted by some miRNAs has been described for psychiatric disorders (Toma et al., 2015; Hauberg et al., 2016) and available evidence supports that the study of common genetic variation across genes targeted by miRNAs dysregulated in different conditions or traits might be useful to disentangle the genetic correlation structure between them. For example, genetic variation within MIR19A/MIR19B has been implicated in the genetic overlap of educational attainment with ASD and attention hyperactivity disorder (ADHD) (Verhoef et al., 2021).

The current study was designed to assess the shared genetic contribution to both ASD and epilepsy within target genes of miRNAs dysregulated in both conditions. To achieve this aim, we first identified the target genes of miRNAs previously described to be altered in both conditions in more than one study. MiRNA target predictions from various *in silico* sources were used to ensure a high consistency of the selected genes. Then, we evaluated partial genetic covariance between ASD and epilepsy across these miRNAs’ targets and compared it against their covariance across genes not targeted by these miRNAs. Given the available evidence indicating shared miRNAs’ dysregulation in both conditions, we hypothesize that common genetic variation within these miRNAs’ targets will inform about the genetic correlation structure between both phenotypes. Finally, using polygenic transmission disequilibrium test (pTDT), a method previously performed in a larger ASD cohort (Weiner et al., 2017), we analyzed ASD and epilepsy polygenic transmission using an ASD cohort of 233 trios and analyzed differences in these polygenic contributions to ASD with and without comorbid epilepsy.

## Methods

### Genetic summary data from previous studies

GWAS summary statistics were used for genetic correlation analysis and polygenic score calculations. GWAS data for ASD (Grove et al., 2019) and epilepsy (Abou-Khalil et al., 2018) used in this study were downloaded from the PGC (https://www.med.unc.edu/pgc/download-results/) and the TILAE (http://www.epigad.org/gwas_ilae2018_16loci.html) consortia repositories.

### ASD sample for polygenic score calculation

Genetic data from a Spanish cohort of ASD trios (comprising subjects with and without comorbid epilepsy; Supplementary Data Sheet S1) was used to evaluate polygenic transmission in an independent sample. Individuals aged three or above with a diagnosis of ASD (N = 233) and their parents were recruited from AMITEA, a specific outpatient ASD program at the Department of Child and Adolescent Psychiatry, Hospital General Universitario Gregorio Marañón in Madrid, Spain. The Research Ethics Committee at Hospital General Universitario Gregorio Marañón reviewed and approved the study. All the participants and/or their legal representatives provided written informed consent after a full explanation of the study procedures. Diagnosis of ASD was established by child psychiatrists with extensive experience in ASD who had completed clinical training in the autism diagnostic interview-revised (ADI-R) and research training in the autism diagnostic observation schedule (ADOS-2). All diagnoses were based on best clinical judgment after full review of all available clinical information following Diagnostic and Statistical Manual of Mental Disorders, Fourth Edition Text Revision or Fifth Edition (DSM-IV-TR or DSM-5) criteria. When necessary (due to inconsistencies found between the sources of information available), the ADOS-2 and/or ADI-R were also administered. ASD trios were later divided in those with (ASD_EPI; N = 36 trios) and without (ASD_NoEPI; N = 197 trios) comorbid epilepsy as per medical records. Available phenotypic information was included in Supplementary Data Sheet S1 **.**

### Selection of miRNAs involved in both ASD and epilepsy pathophysiology

We identified altered miRNAs in both ASD and epilepsy after reviewing available literature. Reporting Items for Systematic Reviews and Meta-analysis (PRISMA) guidelines were followed to ensure the reproducibility of the analyses performed. The first phase consisted in selecting original papers (no reviews or comments) available in PubMed from 2010 to present, published in English, based on human samples, focusing on miRNAs expression levels, and including at least five patients with either ASD or epilepsy and a control group for comparison. The search was performed in January 2022. The search terms are available in Supplementary Data Sheet S2. Two reviewers screened all retrieved papers, independently and in duplicate, in two selection steps: the first consisted of the analysis of the abstracts and the second consisted of the analysis of the whole papers. Any doubt was solved by consensus. Afterward, both reviewers collected independently data from each selected article, extracting information about study characteristics, source of sample (tissue type) and direction of dysregulation (up- or downregulated), as described in Supplementary Data Sheet S3. We did not extract any additional information that goes beyond the aim of the present study. For the subsequent analyses, only those miRNAs whose expression levels significantly differed between cases and controls in both conditions in at least two independent studies were considered (N = 14), in order to ensure the inclusion of miRNAs whose dysregulation had been replicated at least once.

### Prediction of genes targeted by miRNAs involved in both ASD and epilepsy

Target genes of the 14 selected miRNAs were determined with miRNAtap (Pajak and Simpson, 2016) (https://bioconductor.org/packages/release/bioc/html/miRNAtap.html). MiRNAtap is an R package that integrates ranked miRNA target predictions from DIANA, Targetscan, PicTar, Miranda, and miRDB available online and aggregates them with various methods, which improves quality of predictions above any of the single sources (Pajak and Simpson, 2016). A total of 23,476 protein coding genes from ENSEMBL included in HUGO Gene list (HGNC) were considered. We included target genes that appeared as predicted targets in at least four out of the five different sources of *in silico* prediction of miRNA targets. Genes were considered targets for each source when specific threshold were exceeded (Supplementary Data Sheet S4). 4,581 genes were targeted by at least one of the 14 miRNAs studied here.

Additional filters were also performed to restrict target genes to brain expressed genes and haploinsufficient genes (since haploinsufficiency of ASD (Katayama et al., 2016; Yan et al., 2022) and epilepsy (Chénier et al., 2014; Carvill et al., 2021) related genes has been demonstrated as a risk factor). For the first filter, genes were considered brain expressed as expressed in any GTEx v7 brain tissue (N = 18,036 genes). 16,573 out of 23,476 genes were brain expressed. For the second filter, Decipher predictions of haploinsufficiency (https://www.deciphergenomics.org/about/downloads/data; (Huang et al., 2010)) across the human genome (hg19) were downloaded, and those genes belonging to the top 25% with the highest haploinsufficiency score were used as haploinsufficient genes (N = 4,764 genes). 313 out of 23,476 genes belonged to the top 25% genes with the highest haploinsufficiency score. All gene lists are provided in Supplementary Data Sheet S5.

### Genome-wide correlation and partial genetic covariance between ASD and epilepsy

Genome-wide genetic correlation between ASD and epilepsy was evaluated by LD-score regression (LDSC) (Bulik-Sullivan et al., 2015), using the recommended settings (https://github.com/bulik/ldsc). This method generates a score for each SNP, based on the level of linkage disequilibrium with the nearby variants. The z-score of each variant for Trait one is multiplied with the z-score for Trait two and then a regression of this value against LD scores is performed. The coefficient obtained (i.e., the slope) represents the genetic correlation; high values indicate high impact of the SNP on both traits.

GNOVA (Lu et al., 2017) (GeNetic cOVariance Analyzer) (https://github.com/xtonyjiang/GNOVA) was used to estimate genetic covariance between ASD and epilepsy across the following partitions created: A) variants within the 4,581 target genes of any of the 14 selected miRNAs (miR genes) B) variants within the 4,543 target genes of any of the 14 selected miRNAs and expressed in the human brain and C) variants within the 313 target genes belonging to the top 25% percent with highest described haploinsufficiency. Annotation files for these genomic partitions (bed files encompassing whole gene bodies) were created following the recommended settings (https://github.com/bulik/ldsc/wiki/LD-Score-Estimation-Tutorial).

On account of the lower number of genes in miR relative to the 18,895 protein coding genes not targeted by any of the 14 selected miRNA (no_miR genes), estimated genetic covariance between ASD and epilepsy across miR was compared to the distribution of covariances across no_miR from 1,000 random selections of the same number of genes (4,581) from the no_miR annotation.

1,000 Genomes European population-derived reference data was used for LD scores calculation both in LDSC and GNOVA.

### Polygenic scores calculation

The 233 Spanish ASD trios (Supplementary Data Sheet S1) were sequenced as part of the Autism Sequencing Consortium (ASC) (Satterstrom et al., 2020). Exome data was used to calculate polygenic risk scores (PRS) in the 233 Spanish ASD trios. Firstly, exome based VCF files were imputed at the Michigan Imputation Server (https://imputationserver.sph.umich.edu/) using the 1,000 Genome Project phase 3v.5 reference panel to capture genomic variants beyond exome. From 654,286 variants, a total of 38.1 million imputed variants were obtained. Only biallelic variants with imputation quality score >0.9 and minor allele frequency (MAF) > 0.1% were considered (72,292 genotyped and 997,210 imputed SNPs were retained). Then, exome based PRS scores were calculated. The GWAS summary statistics for ASD and epilepsy mentioned above were used as the discovery sample. PRS were calculated for each individual from the target sample as the sum of the number of effect alleles weighted by their effect in the discovery sample. Indels were excluded. Clumping was performed using PLINK v1.9 code “--clump-r2 0.1 --clump-kb 500”. We used Flip Strand and removed ambiguous genomic positions.

For each individual, we initially calculated the following PRS scores:

- ASD PRS based on variants across target genes from any of the 14 selected miRNAs
- Epilepsy PRS based on variants across target genes from any of the 14 selected miRNAs
- ASD PRS based on variants across genes not targeted by any of the 14 selected miRNAs
- Epilepsy PRS based on variants across genes not targeted by any of the 14 selected miRNAs.

In all cases, different PRS were generated across different P thresholds (*p* < 0.00005; 0.001; 0.01; 0.05; 0.1 and 0.2 in the discovery dataset). Then, polygenic transmission disequilibrium tests (pTDT) were performed, using PRS information from the parent-child trios. Briefly, the expected PRS distribution of the offspring is compared with the average PRS distribution of the parents, and their deviations are tested with a one-sample *t*-test. For each disorder and genomic partition, the P threshold with the most significant *p*-value from pTDT in the whole sample was selected. Polygenic transmission of ASD and epilepsy predisposing variation within or outside the miRNA target genes (miR and no_miR) was then calculated in ASD_NoEPI and ASD_EPI subsamples of ASD trios, separately, at the most significant P threshold in each case.

### Statistical analyses

Genetic covariance analyses were performed following the recommended settings by the developers of GNOVA and LDSC. For multiple test comparison, Benjamini–Hochberg FDR correction was performed.

To assess for disequilibrium of polygenic transmission of common predisposing variation to ASD and epilepsy in ASD_NoEPI and ASD_EPI subsamples, one-sided *t*-test were performed in form of pTDT. Full procedure is described in previous work (Weiner et al., 2017). Significant transmissions were confirmed with random permutation of ASD_EPI and ASD_NoEPI subjects.

In case of significant pTDT, comparison of polygenic transmission values between ASD_NoEPI and ASD_EPI groups were performed with two-sample *t*-test. Data normality was contrasted with Shapiro–Wilk test. Equality of variances across groups was assessed with Bartlett test.

## Results

### Target genes of miRNAs dysregulated in ASD and epilepsy

**FIGURE 1:**
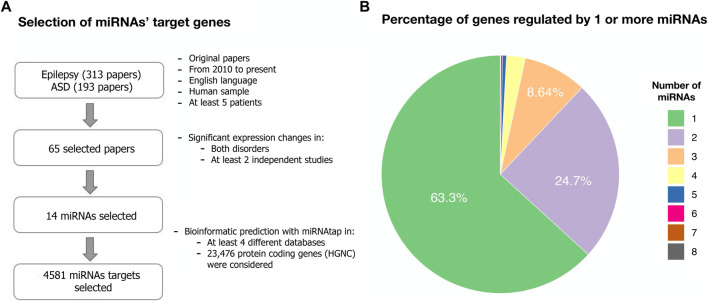
Target genes from dysregulated miRNAs in both ASD and epilepsy. **(A)** Flowchart describing the systematic search for dysregulated miRNAs in both ASD and epilepsy and their target genes (Methods). The search terms and the list of selected papers can be found in Supplementary Data Sheet S2–3. **(B)** Percentage of the 4,581 predicted target genes regulated by one or more of the selected 14 miRNAs.

We searched for miRNAs dysregulated in both ASD and epilepsy. Our systematic search of PubMed produced 313 results for epilepsy and 193 for ASD. A total of 143 miRNAs were dysregulated in either ASD or epilepsy. After screening and selecting papers according to our criteria (see **Methods)**, 14 miRNAs involved in both disorders were selected: let-7, miR-21, miR-27, miR-34, miR-92, miR-124, miR-129, miR-145, miR-146, miR-155, miR-181, miR-193, miR-199, miR-223 (Table 1; Figure 1).

**TABLE 1:** Dysregulated miRNAs in both ASD and epilepsy and number of target genes regulated by each miRNA. The reported miRNAs were identified after a systematic literature search and included all the miRNAs whose expression levels significantly differed between cases and controls in both disorders in at least two independent studies (Figure 1, Supplementary Data Sheet S3). The target genes database was obtained with the miRNAtap R package.

*MicroRNA*	*Dysregulation (ASD)*	*Dysregulation (Epilepsy)*	*Number of target genes*
*let- 7*	Down	Down/Up	396
*miR- 21*	Up	Down/Up	2,563
*miR- 27*	Down	Up	304
*miR- 34*	Up	Down/Up	873
*miR- 92*	Up	Down	505
*miR- 124*	Up	Down/Up	753
*miR- 129*	Up	Up	755
*miR- 145*	Down/Up	Down/Up	46
*miR- 146*	Up	Up	120
*miR- 155*	Up	Up	117
*miR- 181*	Down/Up	Up	225
*miR- 193*	Down	Up	103
*miR- 199*	Up	Up	225
*miR- 223*	Up	Down/Up	41

We considered a total of 4,581 protein-coding target genes of these 14 miRNAs robustly present in various sources from miRNAtap (mIR genes; Table 1). Around 63% of genes were regulated by only one from the 14 miRNAs selected (Figure 1).

### Genetic correlation and partial covariance between ASD and epilepsy

**FIGURE 2:**
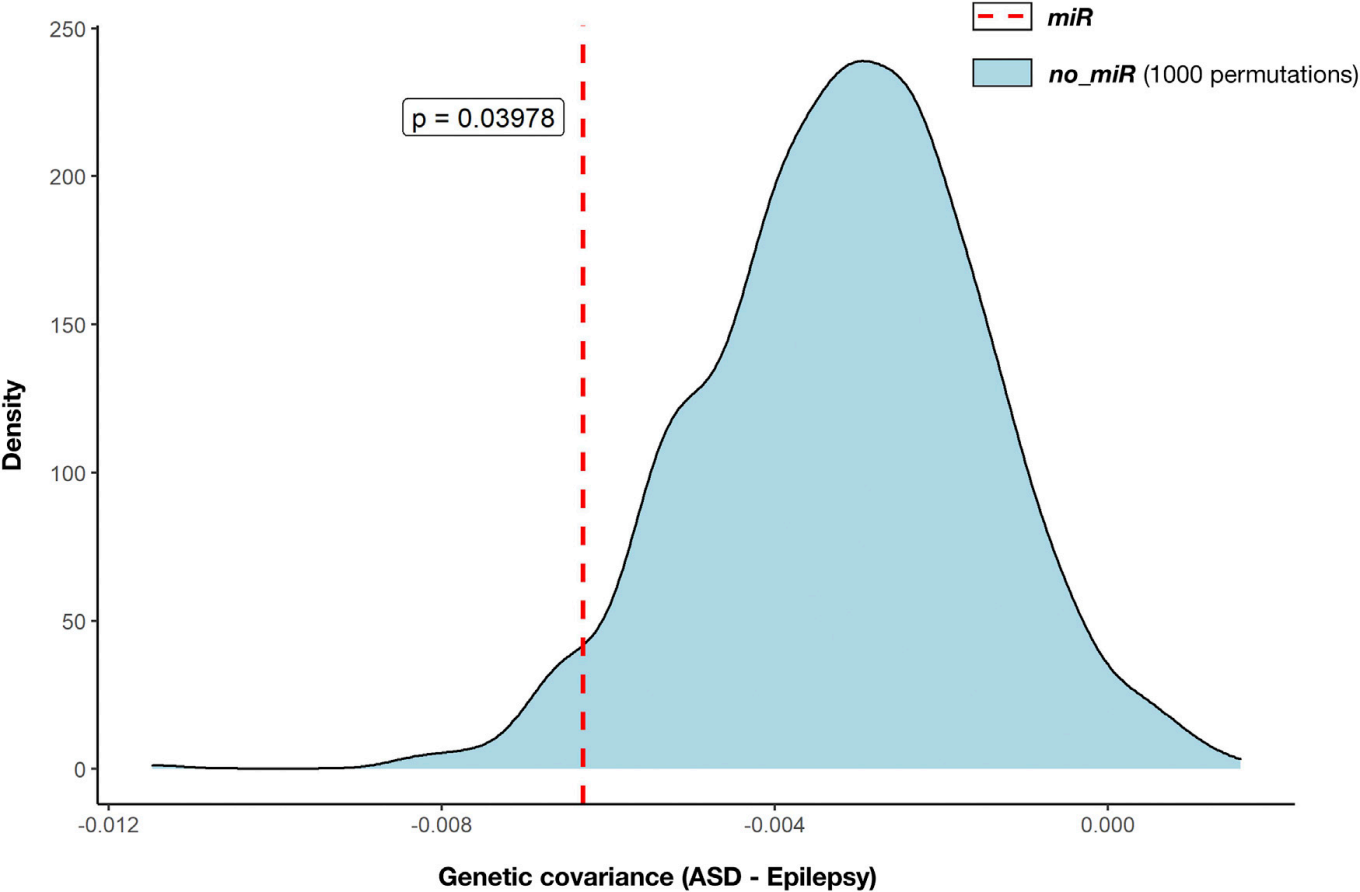
Genetic covariance between autism (ASD) and epilepsy across miRNA target gene annotations. Partial genetic covariances were calculated with GNOVA (Table 2). Estimated genetic covariance between ASD and epilepsy across genes targeted by the selected 14 miRNAs (miR: 4,581 genes, red dashed line) was compared against 1,000 genetic covariances across genes not targeted by the selected 14 miRNAs (no_miR, blue density distribution) that were estimated after 1,000 random selections of the same number of genes (4,581 genes). Statistical comparison between genetic covariance across miRNA target genes and the distribution of genetic covariances across the remaining genes was performed by one-sided *t*-test. The derived *p*-value is displayed (*p* = 0.040).

We used GNOVA to assess the partial genetic covariance between ASD and epilepsy across variants within genes targeted by the 14 selected miRNAs (N_Genes_ = 4,581). Negative covariance within miR genes was observed (rho (95%CI) = -0.006 (-0.012; -0.001); *p* = 0.0228; Figure 2; Supplementary Data Sheet S6). Genetic covariance between ASD and epilepsy at the whole genome was also negative but not significant (rho (95%CI) = -0.021 (-0.04; 0.003); *p* = 0.069; Supplementary Data Sheet S6).

Moreover, negative genetic covariance between ASD and epilepsy was greater across mIR genes than the distribution of 1,000 estimated covariances calculated by using the same number of genes (4,581) randomly selected from the remaining 18,895 not targeted by the miRNAs (no_miR genes). Genetic covariance within miR was significantly smaller than the distribution of covariances based on no_miR genes (*p* = 0.039; Figure 2).

The genetic covariance between ASD and epilepsy after restricting to miR genes expressed in the human brain (N_Genes_ = 4,544) was also significant (rho (95%CI) = -0.005 (-0.009; -0.001); *p* = 0.0248; Supplementary Data Sheet S6). Interestingly, when restricting to haploinsufficient miR genes (N_Genes_ = 313), positive covariance was observed (rho (95%CI) = 0.009 (0.003; 0.016); *p* = 0.0102; Supplementary Data Sheet S6. However, the number of miRNA targets across haploinsufficient genes was significantly lower than across non-haploinsufficient genes (Chi-square X^2^ = 402.95; *p* < 10^−16^), suggesting underrepresentation of haploinsufficient genes across miRNA targets.

### Polygenic score prediction in the ASD sample with and without comorbid epilepsy

We then calculated ASD and epilepsy polygenic risk scores (PRS) in our cohort of ASD complete trios. PRS were calculated for variants across genes targeted and non-targeted by the 14 miRNAs selected. Transmission of predisposing variants in each case (pTDT) was assessed for ASD trios including individuals with (ASD_EPI; N = 36) and without (ASD_NoEPI; N = 197) comorbid epilepsy.

**FIGURE 3:**
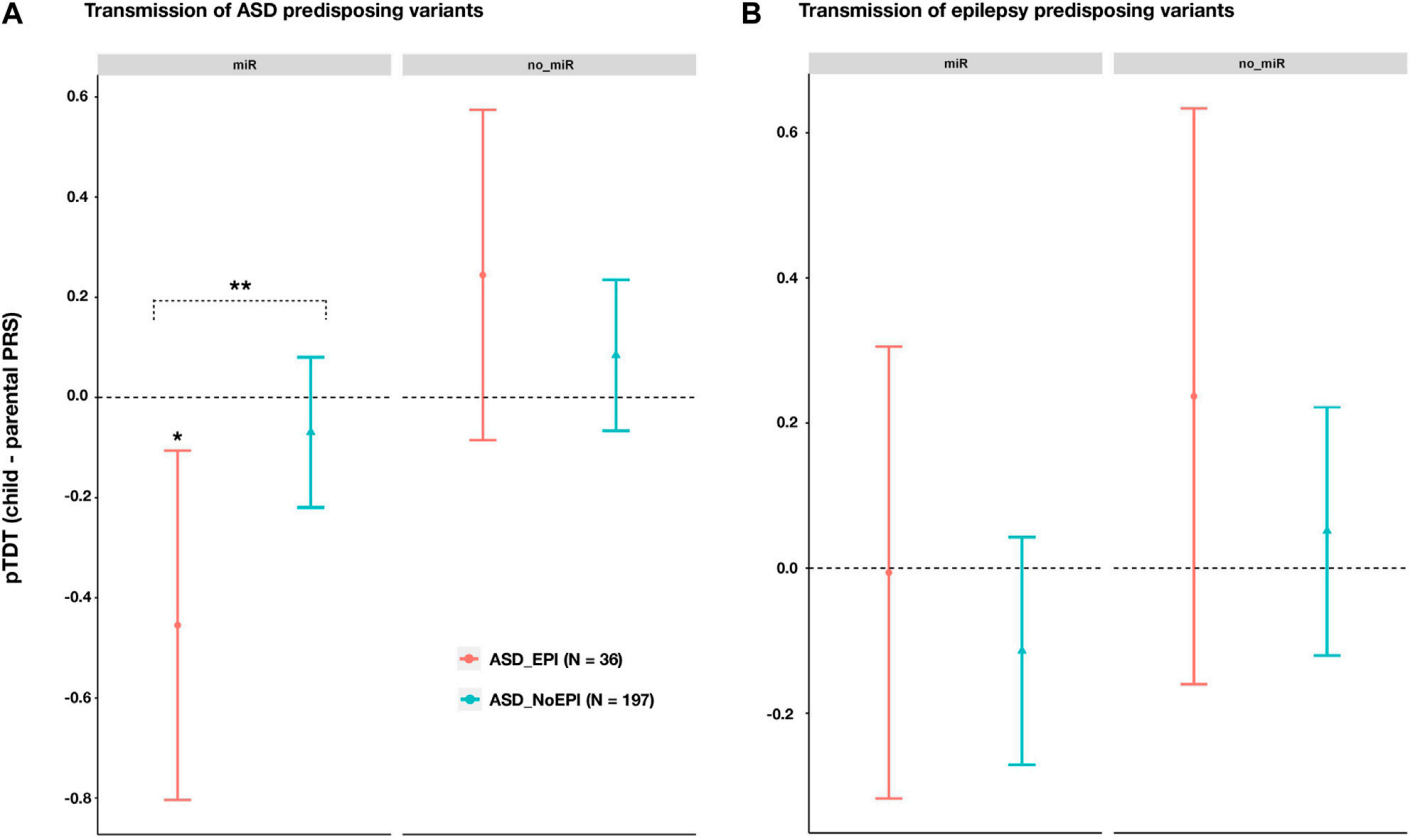
Polygenic transmission disequilibrium test (pTDT) on ASD and epilepsy risk variants in ASD trios with (ASD_EPI trios, N = 36), and without (ASD_NoEPI trios; N = 197) comorbid epilepsy. Two-tailed t-tests were performed to test for over or under-transmission of common predisposing alleles to ASD **(A)** or epilepsy **(B)**, from parents to children (Supplementary Data Sheet S7). Common predisposing variation across genes targeted (miR; N = 4,581 genes) or not targeted (no_miR; N = 18,895 genes) by the 14 selected miRNAs. Error bars represent 95% confidence intervals. For each disorder and genomic partition, we selected the most significant *p*-value threshold from the pTDT previously performed in the whole sample of ASD trios (Supplementary Data Sheet S7). **(A)** pTDT on ASD predisposing variants **(B)** pTDT on epilepsy predisposing variants. Significant *p*-values from permutation analyses are marked with an asterisk. two sample *t*-test was performed to evaluate differences in pTDT between ASD_EPI and ASD_noEPI subsamples.

Across miRNA target genes, we observed a lower transmission of ASD risk alleles than expected by chance for ASD_EPI trios (pTDT (95%CI) = -0.4549 (0.1060; -0.8039); *p* = 0.0121; Figure 3A), but not for ASD_NoEPI trios (pTDT (95%CI) = -0.0697 (0.0802; -0.2197); *p* = 0.3601; Figure 3A). We confirmed these results by conducting 10,000 random permutations of comorbid epilepsy status (ASD_EPI-P_perm_ = 0.0153; ASD_ NoEPI -P_perm_ = 0.8060). Difference in ASD polygenic transmission within miRNA target genes between both groups was also found (two sample t = 2.0496, *p* = 0.0457; Supplementary Data Sheet S7).

Across genes not targeted by the selected 14 miRNAs, we observed no significant transmission disequilibrium of ASD risk alleles in either subgroup (ASD_EPI: pTDT (95%CI) = 0.2445 (0.5744; -0.0855) *p* = 0.1415; ASD_NoEPI: pTDT (95%CI) = 0.0841 (0.2347; -0.0664); *p* = 0.2719; Figure 3A).

No significant differences were found in transmission of epilepsy risk variants, neither between the ASD_EPI and ASD_NoEPI nor between the miR and no_miR genomic partitions (Figure 3B **;** Supplementary Data Sheet S7).

## Discussion

This study suggests the presence of a negative genetic relationship between ASD and epilepsy across common genetic variation within target genes of miRNAs involved in both conditions. Moreover, we described a lower transmission of ASD risk alleles from variants within target genes of these miRNAs in ASD patients with comorbid epilepsy than expected by chance. Our results support the idea of a genetic divergence between ASD and epilepsy in terms of common variation, which is more marked when analyzing pathways potentially involved in both conditions such as some miRNA regulatory networks. These results contrast with the direct genetic overlap found between both phenotypes across rare *de novo* disrupting variation and contribute to conceptualizing the complexity of the genetic relationships between these conditions.

From the genomic perspective, while rare *de novo* variation affecting neurodevelopmental related genes has been previously described as a predisposing factor to both ASD and epilepsy (Todd and Bassuk, 2018; Hiraide et al., 2019; Mahjani et al., 2021), no clear link between both disorders at the level of common genetic variation has been found (Abou-Khalil et al., 2018; The Brainstorm Consortium et al., 2018). This is important since most ASD genetic risk resides in common polygenic contribution (Gaugler et al., 2014). Here in this study, by restricting our analysis to the regulatory networks of 14 miRNA involved in both ASD and epilepsy, we described a negative genetic correlation between both disorders. Although these results may appear counterintuitive, since these 14 miRNAs have been described to be affected in both phenotypes, several recent studies have described patterns of genetic relationships between ASD and comorbid phenotypes at the level of common genetic variation that differ from those found for disrupting rare variation. For instance, whereas ASD subjects with low IQ have a significantly increased number of disrupting *de novo* mutations (Iossifov et al., 2014), a positive genetic correlation between ASD and IQ has been described (Zhang et al., 2021). Moreover, ASD polygenic scores predict higher cognitive performance in the global population (Clarke et al., 2016). Another rare-common disparity in genetic relationships was described for schizophrenia; while there is a consistent overlap across genes disrupted by *de novo* rare variation in schizophrenia and ASD (Kenny et al., 2014; Satterstrom et al., 2020; Singh et al., 2020), shared common variation between both phenotypes has been reported to be chiefly restricted to high functioning autism (González-Peñas et al., 2020).

The negative significant covariance reported here between ASD and epilepsy at the selected miRNA regulatory pathways depict a scenario in which common predisposing variation within miRNA contributes to both conditions in opposite directions. This could be due to the fact that common ASD predisposing variation underpin high functioning autism (González-Peñas et al., 2020) while rare variation is mainly involved in more severe forms of ASD, which are more frequently associated with intellectual disability and epilepsy (Strasser et al., 2018). In this sense, when considering haploinsufficient genes, which have been related with both ASD and epilepsy related genes in terms of rare genetic variation (Katayama et al., 2016; Carvill et al., 2021), we described a clear underrepresentation across target genes of the miRNAs here studied. Also, across the reduced set of haploinsufficient target genes (*n* = 313), a positive correlation between ASD and epilepsy was described, reinforcing the existence of different genetic contributions from genes primarily affected by rare or common genetic variation.

The lower transmission of ASD risk variants that we observed in ASD patients with comorbid epilepsy supports the idea of common variation differently contributing to phenotypic variation across the autism spectrum (Heyne et al., 2018; González-Peñas et al., 2020; Zhang et al., 2021).

In a recent study, Antaki et al. (2022). have described a negative correlation between transmitted ASD PRS and *de novo* protein truncating mutations, consistent with a liability threshold model by which the genetic load needed to meet a diagnostic criterion is reached by the additive contribution of both polygenic background and rare high impact genetic variation, in combination to environmental risk factors (Falconer, 1967). Our results describing a lower ASD PRS transmission in ASD patients with epilepsy than expected by chance suggests this liability threshold model is also observable at a pathway specific level. Therefore, focusing on shared altered pathways between ASD and comorbid conditions such as miRNAs may help to disentangle the genetic variation underlying the observed phenotypic heterogeneity of ASD.

All these findings support that the diagnostic category ASD, as described in the fifth edition of the Diagnostic and Statistical Manual of Mental Disorders (DSM-5) could encompass distinct genetically divergent subcategories (Li et al., 2019) that reflect phenotypic variation within the spectrum. Our results support the observed heterogeneity of rare and common variation contribution to complex disorders and highlight the likely underlying genetic divergence between ASD with and without comorbid epilepsy (Burgess et al., 2019). These results contribute to a better understanding of the biological characteristics of the ASD clinical subtypes and may guide patient stratification by focusing on specific pathways or mechanisms of gene expression regulation.

Our work was subject to several limitations. First, the discovery sample size of the latest GWAS of epilepsy used here was modest. Since various types of conditions exist under the epilepsy umbrella, larger and stratified case-control cohorts would be required to reach sufficient power and comparable to ASD and to validate the results presented here. Second, our independent cohort for pTDT analyses, although carefully phenotyped, had also a limited sample size. These findings need to be replicated in a larger sample. Finally, we here selected a list of miRNAs for which dysregulation is observed in both conditions through a comprehensive literature search. Although we only considered miRNAs with significant findings in at least two independent studies, there was heterogeneity among studies in terms of the technique performed and tissue used for the analysis and there could also be miRNA selection or reporting biases. *In vivo* studies incorporating more reliable methods that enable direct detection of miRNA targets could lead to more powerful outcomes.

In summary, our results provide further evidence about the genetic complexity of ASD, suggesting miRNA dysregulation as one important pathway to explain the genetic complexity in the relationship between ASD and epilepsy. A clear understanding of ASD genetic heterogeneity could be useful to identify risk groups and trajectories and pave the way for precision medicine approaches to neurodevelopmental conditions (Table 2).

**TABLE 2:** Genetic covariance analysis between Autism and Epilepsy across whole genome and within target genes of miRNAs dysregulated in both conditions. Whole genome and partial covariances were performed with LD Score Regression (LDSC) and GNOVA, respectively, following the recommended settings (see methods). *p*-values were corrected with Benjamini–Hochberg FDR to control for multiple testing. Brain expressed genes in any GTEx v7 brain tissue were considered in “brain expressed” annotation. Genes belonging to the top 25% with the highest haploinsufficiency score from Decipher predictions were used as haploinsufficient genes.

Annotation	Genetic covariance (SE)	Genetic correlation (SE)	*p*-value	FDR-p
Whole Genome	-0.0216 (0.0121)	-0.1432 (0.0788)	0.069	0.069
mIR genes	-0.00627 (0.00275)	-0.2161 (0.0339)	0.0228	0.0331
mIR genes (brain expressed)	-0.0048 (0.0021)	-0.1984 (0.0384)	0.0248	0.0331
mIR genes (top 25% Haploinsuficient)	0.0092 (0.0033)	0.2202 (000,043)	0.0102	0.0331

## Data Availability

The datasets presented in this study can be found in online repositories. The names of the repository/repositories and accession number(s) can be found in the article/Supplementary Material.
